# HLA predictions from the bronchoalveolar lavage fluid and blood samples of eight COVID-19 patients at the pandemic onset

**DOI:** 10.1093/bioinformatics/btaa756

**Published:** 2020-08-27

**Authors:** René L Warren, Inanç Birol

**Affiliations:** Genome Sciences Centre, BC Cancer, Vancouver, BC V5Z 4S6, Canada; Genome Sciences Centre, BC Cancer, Vancouver, BC V5Z 4S6, Canada

## 1 Introduction

Severe acute respiratory syndrome (SARS)-CoV-2 infections have reached global pandemic proportions in early 2020, affecting over 21 M people worldwide (as of this writing in August 2020; Source: Johns Hopkins University) and showing no signs of easing, except in a few jurisdictions where strict quarantine measures were implemented early on. The resulting coronavirus disease (COVID-19) has a relatively high (∼3.4%) mortality rate (Rajgor *et al.*, 2020)—a figure that varies widely between jurisdictions due to factors yet to be determined. Currently, no vaccines or effective treatments are available. Most current data analysis efforts are, understandably, focussed on the virus itself for the purpose of vaccine development and tracking its evolution for diagnostics and infection monitoring purposes.

Curiously, it is estimated that as high as 18–30% or more of the population may be asymptomatic to SARS-CoV-2 infections (Mizumoto *et al.*, 2020; Nishiura *et al.*, 2020), while other affected individuals exhibit mild to severe to critical symptoms of infection. Thus, gaining insights on host susceptibility to the coronavirus is clearly another important aspect that needs to be worked on and understood (Shi *et al.*, 2020).

One would expect a link between host immunity genes and susceptibility or resistance to infection. The Human Leukocyte Antigen (HLA) gene complex includes two classes of such genes, which encode the Major Histocompatibility Complex (MHC). Proteins of the MHC present (class I) internally- or (class II) externally-derived antigenic determinants (epitopes) to T cells, which upon recognition of the epitope-complex, will mount an immune response to defend against viral and bacterial infections. HLA genes are, therefore, cornerstone to acquired immunity in humans. HLA alleles have also been shown to be factors in susceptibility or resistance to certain diseases, and their frequency and composition in human populations vary widely (http://allelefrequencies.net/). A previous study found HLA class I (HLA-I) genes HLA-B*46:01 and HLA-B*54:01 to be associated with the 2003 SARS coronavirus infections in Taiwan (Lin *et al.*, 2003)—a related disease to the current pandemic.

For over a decade, high-throughput transcriptome sequencing has proven a worthy instrument for measuring changes of gene expression in human diseases and beyond (Wang *et al.*, 2009). Transcriptome analysis has the potential to reveal key genes that are modulated in response to infections, but also has the potential to reveal the HLA composition of affected individuals. A few years ago, we developed an approach for mining high-throughput next-generation shotgun sequencing data for the purpose of HLA determination (Warren *et al.*, 2012), which has since been applied in a broader clinical context (Brown *et al.*, 2014).

Here, we report our initial observations based on transcriptome sequencing (RNA-Seq) libraries prepared from the bronchoalveolar lavage (BAL) fluid and peripheral blood mononuclear cell (PMBC) samples of five and three COVID-19 patients at the early stage of the pneumonia coronavirus outbreak in Wuhan, China, respectively (Xiong *et al.*, 2020; Zhou *et al.*, 2020) (see Section 2). Of note, we identified the HLA-I group A allele A*24:02 in four out of five individuals from the first cohort and class II haplotype DPA1*02:02-DPB1*05:01 in seven out of eight individuals from both cohorts.

## 2 Materials and methods

We downloaded MGISEQ-2000RS paired-end (150 bp) RNA-Seq reads from libraries prepared from the BAL fluid samples of five patients [https://www.ebi.ac.uk/ena/browser/view/PRJNA605983 Accessions: SRX7730880-SRX7730884 denoted in the tables as Patients 1–5, respectively (Zhou *et al.*, 2020)] and BGISEQ-500 paired-end (100 bp) RNA-Seq reads derived from the PMBC samples of three COVID-19 patients from a different study [Run accessions: CRR119891-3 from BIG Data Center (https://bigd.big.ac.cn/) project CRA002390 denoted in the tables as Patients 6–8, respectively (Xiong *et al.*, 2020)]. We note that these are metatranscriptomic RNA samples prepared for the primary purpose of identifying/characterizing the novel coronavirus and identifying host response genes. On each dataset, we ran HLAminer (Warren *et al.*, 2012) in targeted assembly mode with default values (v1.4; contig length ≥200 bp, seq. identity ≥99%, score ≥1000, *e*-value ≥25), predicting HLA-I and class II (HLA-II) alleles and report 4-digit (HLA allele/protein) resolution when top-scoring predictions are unambiguous. Otherwise the 2-digit (allele group) resolution is reported. We also ran HLA prediction software seq2HLA (Boegel *et al.*, 2012; v2.3), OptiType (Szolek *et al.*, 2014; v1.3.4) and arcasHLA (Orenbuch *et al.*, 2020; v0.2.0 with latest code commit 301085e) on the RNA-Seq data derived from the BAL samples of COVID-19 patients (Supplementary Methods). The tool used to perform the reported analysis results in Tables 1 and 2, HLAminer, is available from https://github.com/bcgsc/hlaminer. Predictions are available for download from https://www.bcgsc.ca/downloads/btl/SARS-CoV-2/BAL.

## 3 Results and discussion

We predicted and compiled the likely HLA-I (Table 1) and HLA-II (Table 2) alleles of eight patients at the early stage of the COVID-19 outbreak in Wuhan, China. In the first cohort comprised of five patients, although the BAL fluid samples were initially utilized to identify and characterize the novel coronavirus [Zhou *et al.* (2020) with similar justification in Wu *et al.* (2020)], BAL metagenomics samples are expected to contain host cells/nucleic acids (DNA/RNA). Because HLA genes are expressed at the surface of all human nucleated cells, RNA-Seq data can be employed to determine HLA profiles from BAL samples.

**Table 1.** HLA-I predictions from the BAL fluid samples of five patients at the early stage of the Wuhan seafood market pneumonia coronavirus outbreak and from the PBMC samples of three COVID-19 patients from a different cohort/study

BAL samples from five Wuhan COVID-19 patients					PBMC samples from three Wuhan COVID-19 patients		
Patient 1	Patient 2	Patient 3	Patient 4	Patient 5	Patient 6	Patient 7	Patient 8
A*01:01	A*30:01	A*24:02	A*24:02	A*29	A*02:01	A*01:01	A*02:01
A*24:02	A*02:06	A*26:01	A*02:06	A*24	A*33:03	A*02:03	A*11:01
B*35/B*57	B*51:01	B*15:01	B*40:01	B*54:01	B*56:01	B*46:77	B*56:01
B*48	B*13:02	B*51:01	B*13:01	B*07:05	B*58:01	B*56:03	B*15:438
C*08:72	C*14:02	C*15:02	C*04:03	C*15	C*03:02	C*07:02	C*15:02
C*06:02	C*06:02	C*03:03	C*03:04	—	C*01:02	C*01:02	C*08:01

*Note*: Highest-scoring HLAminer predictions are shown for each HLA-I genes A, B and C. Missing class I genes or (—) denote the absence of a second prediction. Common HLA alleles between two or more patients of a given cohort are highlighted in bold. Ambiguous predictions are shown at the group (2-digit) resolution.

**Table 2.** HLA-II predictions from the BAL fluid samples of five patients at the early stage of the Wuhan seafood market pneumonia coronavirus outbreak and from the PBMC samples of three COVID-19 patients from a different cohort/study

BAL samples from five Wuhan COVID-19 patients					PBMC samples from three Wuhan COVID-19 patients		
Patient 1	Patient 2	Patient 3	Patient 4	Patient 5	Patient 6	Patient 7	Patient 8
DPA1*02:02	DPA1*02:01	DPA1*02:02	DPA1*02:02	DPA1*04:01	DPA1*02:02	DPA1*02:01	DPA1*02:02
—	DPA1*02:02	—	—	—	DPA1*01:03	DPA1*02:02	—
DPB1*05:01	DPB1*13:01	DPB1*05:01	DPB1*05:01	—	DPB1*05:01	DPB1*05:01	DPB1*05:01
DPB1*02:02	DPB1*05:01	—	—	—	DPB1*04:01	DPB1*17:01	—
DQA1*01:03	DQA1*01:02	DQA1*03:01	DQA1*01:01	—	DQA1*05:01	DQA1*03:01	DQA1*06:01
DQA1*05	DQA1*02:01	—	DQA1*05:01	—	—	DQA1*01:02	DQA1*01:02
DQB1*03:01	DQB1*05:02	DQB1*04:01	DQB1*03:01	—	DQB1*03:02	DQB1*02:01	DQB1*03:01
DQB1*06:01	DQB1*02:01	DQB1*03:02	—	—	DQB1*02:01	DQB1*03:03	DQB1*06:01
—	DRB4*01:01	DRB4*01:01	—	—	DRB4*01:01	DRB4*01:01	—
—	—	—	—	—	—	—	—

*Note*: Highest-scoring HLAminer predictions are shown for HLA-II genes DPA1, DPB1, DQA1, DQB1 and DRB4. Missing class II genes or (—) denote the absence of predictions. Common HLA alleles between two or more patients of a given cohort are highlighted in bold. Ambiguous predictions are shown at the group (2-digit) resolution.

We observe the HLA-A*24:02 allele in four out of five (80%) patients of the first cohort, but this allele was not predicted in the second cohort whose SARS-CoV-2 positive patients were also admitted in a Wuhan hospital (Table 1) (Xiong *et al.*, 2020). In the absence of an equivalent BAL metatranscriptomic dataset with known HLA genotypes, we opted to run the established prediction software seq2HLA (Boegel *et al.*, 2012) and OptiType (Szolek *et al.*, 2014) and the recently published utility arcasHLA (Orenbuch *et al.*, 2020), in an effort to help validate the predictions we obtained from HLAminer on the BAL cohort RNA-Seq (Supplementary Table S1). We observe good concordance among the tools, with the highest concordance observed between HLAminer and seq2HLA when predicting HLA-I gene A (100% concordance) and between HLAminer and OptiType when predicting HLA-I B and C genes (100% and 88.9%, respectively; Supplementary Table S1). We note that arcasHLA failed to output predictions altogether on these metatranscriptomic data, likely due to low HLA signal. Of interest, the HLA-I allele A*24:02 prediction that we report in Table 1 was recapitulated with seq2HLA. HLA-A*24 is a common group of alleles in South-Eastern Asian populations, and the frequency of the HLA-A*24:02 allele can be high, especially in indigenous Taiwanese populations, reaching as high as 86.3% (http://allelefrequencies.net/). It has been reported that the five patients from the first cohort were sellers and delivery workers at the Wuhan seafood market, but since no information on patient ethnicity/ancestry is available (Zhou *et al.*, 2020), no inferences can be made with respect to population frequency, especially given the small sample size. Also, of note, we observe the HLA-II DPA1*02:02 and DPB1*05:01 haplotype predicted in seven out of the eight (87.5%) patients (Table 2).

We point out that HLA-A*24 has not been previously reported as a risk factor for SARS infection (Sun and Xi, 2014). There are reports of other disease association with HLA-A*24:02, notably with diabetes (Adamashvili *et al.*, 1997; Kronenberg *et al.*, 2012; Nakanishi and Inoko 2006; Noble *et al.*, 2002), which is a recorded potential risk factor in COVID-19 patients (Guan *et al.*, 2020). Both DPA1*02:02 and DPB1*05:01 occur at relative high frequency (44.8% and 31.3%, *n*=1490) in Han Chinese (Chu *et al.*, 2018), and associations of those particular type II alleles with narcolepsy (Ollila *et al.*, 2015) and Graves’ disease (Chu *et al.*, 2018), both autoimmune disorders, have been reported in that population. Further, a genome-wide association study found a link between HLA-DPB1*05:01 and chronic hepatitis B in Asians, and it has been suggested that this risk allele may impact one’s ability to clear viral infections (Kamatani *et al.*, 2009; Ollila *et al.*, 2015). HLA also informs vaccine development. This knowledge would help prioritize SARS-CoV-2 derived epitopes predicted to be stable HLA binders (Kiyotani *et al.*, 2020; Nguyen *et al.*, 2020; Prachar *et al.*, 2020; Yarmarkovich *et al.*, 2020). HLA-I A*24:02 was reported to be among just a few allotypes that showed stable binding with more than 10 epitopes derived from the SARS-CoV-2 proteome (Kiyotani *et al.*, 2020; Prachar *et al.*, 2020). In contrast, previously reported SARS risk allele HLA-B*46:01 (Lin *et al.*, 2003) had amongst the fewest number of predicted binding SARS-CoV-2 peptides (Nguyen *et al.*, 2020).

Further research into host susceptibility and resistance to SARS-CoV-2 infections on larger population cohorts and from different jurisdictions is sorely needed as it may help us better manage and mitigate risks of infections. We stress that our observations were derived from small sample sets, and caution that host susceptibility gene inferences require larger cohorts and properly designed data collection experiments with controls, to help quantify the false positive rate and confidence in predictions. Our letter highlights the technical feasibility and challenges associated with deriving HLA types directly from metatranscriptomic RNA-Seq libraries prepared from COVID-19 patient samples and not collected specifically for that purpose. We chose to communicate our early findings in this domain to facilitate rapid development of response strategies.

## Funding

This work was supported by Genome BC and Genome Canada [281ANV]; and the National Institutes of Health [2R01HG007182-04A1]. The content of this paper is solely the responsibility of the authors, and does not necessarily represent the official views of the National Institutes of Health or other funding organizations. 

*Conflict of Interest*: none declared.

## Supplementary Material

btaa756_Supplementary_Data
