# Supplementary material for: HLA predictions from the bronchoalveolar lavage fluid and blood samples of eight COVID-19 patients at the pandemic onset
Source: Bioinformatics. 2020 Aug 27;36(21):5271–3. doi: 10.1093/bioinformatics/btaa756 (PMC7540287; doi:10.1093/bioinformatics/btaa756)
Supplement: btaa756_Supplementary_Data [file btaa756_supplementary_data.docx]

Supplementary Material for: **Warren and Birol (2020) HLA predictions from the bronchoalveolar lavage fluid and blood samples of eight COVID-19 patients at the pandemic onset**

**Supplementary Method**

We ran the following HLA prediction methods, with options: seq2HLA (Boegel et al., 2012; v2.3; options: -1 fastq1 -2 fastq2 -p 48), OptiType (Szolek et al., 2014; v1.3.4, options: razers3 -i 95 -m 1 -dr 0 -o outhla1.bam hla_reference_rna.fasta fastq1, samtools bam2fq outhla1.bam > outhla1.fastq, same razers3 option on fastq2 followed by OptiTypePipeline.py -i outhla1.fastq outhla2.fastq --rna -v -o optitypeOUT) and arcasHLA (Orenbuch et al., 2020; v0.2.0 with latest commit 301085e; options: genotype outhla1.fastq outhla2.fastq -g A,B,C,DPA1,DPB1,DQA1,DQB1 -o arcasHLA -t 8 -v, followed by: partial outhla1.fastq outhla2.fastq -g A,B,C,DPA1,DPB1,DQA1,DQB1 -G arcasHLA/outhla1.genotype.json -o arcasHLA -t 8 -v ).

Raw HLA prediction output files from all tools are available at:

https://www.bcgsc.ca/downloads/btl/SARS-CoV-2/BAL

**Supplementary Table S1**. Concordance of HLA allele calls between HLAminer, seq2HLA and OptiType on RNA-Seq derived from the BAL samples of COVID-19 patients*. Bold face figures denote the highest concordance achieved between pair of tools on a specific HLA class I gene.

| **Genes** | **HLAminer v. seq2hla** | **HLAminer v. OptiType** | **seq2HLA v. OptiType** |
| --- | --- | --- | --- |
| A | **100.0%** | 80.0% | 70.0% |
| B | 90.0% | **100.0%** | 90.0% |
| C | 77.8% | **88.9%** | 80.0% |

*arcasHLA also ran on those samples and it failed to produce HLA predictions, likely due to low signal / HLA sequence coverage, as indicated in its output: “Not enough reads aligned to genotype”.
